# An investigation of the prevalence of impacted teeth in Kabul, Afghanistan: A retrospective cross-sectional study

**DOI:** 10.1097/MD.0000000000047917

**Published:** 2026-03-06

**Authors:** Shahab Uddin Ahmadi, Hedayatullah Ehsan, Yahya Fayaz, Sara Wali, Reza Fahimi, Musa Joya, Tamana Barakati, Omid Humkar, Hassina Shadab, Ismail Yaqubi

**Affiliations:** a Department of Stomatology, Khatam-Al-Nabieen University, Kabul, Afghanistan; b Clinical Department of Dentistry, Ghalib University, Kabul, Afghanistan; c Department of Stomatology, Khatam-Al-Nabieen University, Kabul, Afghanistan; d Department of Stomatology, Zawul University, Kabul, Afghanistan; e Department of Clinic, Kateb University, Kabul, Afghanistan; f Department of Physics, University of Surrey, Surrey, UK; g Department of Medicine and Dentistry, Queen Mary University of London, London, UK; h Department of Stomatology, Noman Sadat Institute of Higher Education, Kabul, Afghanistan; i Department of Periodontology, Kabul University of Medical Science, Kabul, Afghanistan; j Department of Oral and Maxillofacial Surgery, Stomatology National Curative and Specialized Hospital, Kabul, Afghanistan.

**Keywords:** Afghanistan, dental epidemiology, impacted teeth, Kabul, mandibular third molars, prevalence, retrospective study, tooth impaction

## Abstract

Impacted teeth represent a significant dental health concern with potential complications including infections, cysts, and crowding. While extensively studied globally, no prior research has examined prevalence patterns in Afghanistan’s clinical population. This study aimed to determine the clinical prevalence and demographic patterns of impacted teeth among patients treated at Kabul National Curative and Specialized Stomatology Hospital. In this retrospective study, we analyzed 4000 patient records (2019–2022), identifying 298 cases with radiographically confirmed impacted teeth. Data on demographics, impaction types, and associated factors were evaluated using descriptive statistics and multivariate logistic regression. The clinical prevalence of impacted teeth was 7.5% (298/4000). Mandibular third molars were most common (65.1%), showing significantly higher prevalence in males (OR = 1.9, *P* = .002) and patients aged 21 to 30 years (OR = 3.2, *P* <.001). Maxillary canines accounted for 18.5% of cases with no gender disparity (*P* = .12). The median age was 24 years with nearly equal gender distribution (48.7% male, 51.3% female). This first Afghan study reveals distinct impaction patterns, particularly the predominance of mandibular third molars in young adult males. Findings emphasize the need for targeted prevention and early intervention strategies in clinical practice, while highlighting the necessity for population-based studies to establish national prevalence.

## 1. Introduction

Tooth impaction, where teeth fail to erupt in the dental arch due to inadequate space, abnormal positioning, or atypical growth processes, is a common dental issue.^[1]^ Impacted teeth, also known as unerupted teeth, can result from various local and systemic conditions.^[1,2]^ Local factors include cleft palate and cleft lip (CP + L), insufficient dental arch length, malposition, odontogenic tumors, prolonged retention of primary teeth, and supernumerary teeth.^[3]^ Systemic factors, though less common, include febrile conditions, cleidocranial dysplasia, Down syndrome, and endocrine disorders.^[1,4]^

The prevalence and distribution of impacted teeth vary across populations and ethnic groups, influenced by factors such as age, ethnicity, and eruption timing.^[4]^ Maxillary canines and third molars are the most frequently impacted teeth, followed by mandibular wisdom teeth, largely because third molars are the last to erupt in the oral cavity and often lack sufficient space.^[2–5]^ These variations necessitate population-specific studies to understand the regional patterns and implications of tooth impaction.^[6]^

Tooth impaction can cause a range of complications, including tooth movement, appearance issues, and functional problems.^[6]^ Accurate diagnosis and management are crucial for both patients and clinicians. Advanced imaging techniques like computed tomography and panoramic radiography are essential for pinpointing the exact location of impacted teeth, aiding in effective diagnosis and treatment.^[6,7]^ Panoramic radiographs are often the first choice as they provide comprehensive details about the teeth and surrounding structures.^[7–9]^

The American Association of Oral and Maxillofacial Surgeons recommends early extraction of impacted teeth to prevent complications such as decay, cyst or tumor formation, periodontal disease, and odontogenic infections.^[10–15]^ This study aims to assess the prevalence and pattern of tooth impaction in Kabul, Afghanistan, considering patients’ age, gender, and medical history. By filling the gap in regional data, this study contributes to the broader understanding of tooth impaction’s clinical significance and aids in the development of targeted preventive measures and interventions.

## 2. Materials and methods

Of 4000 patients screened between 2019 and 2022, 298 had radiographically confirmed impactions (7.5% prevalence). As all patient records during the study period were included, no prior sample size calculation was required. This comprehensive enumeration approach ensures full coverage of the study population. As this study employed a retrospective design, all patient records available during the defined study period (March 2019 to August 2022) at the National Curative and Specialized Stomatology Hospital were screened. Therefore, no a priori sample size calculation was performed. A total of 4000 records were reviewed, and 298 cases with radiographically confirmed impacted teeth were included based on eligibility criteria. While hospital-based sampling may overestimate prevalence versus population studies, it provides critical baseline data for Kabul clinical population. The National Curative and Specialized Stomatology Hospital provides treatment services for orofacial diseases for many people throughout Afghanistan. Prior to the commencement of our study, rigorous scrutiny and approval were secured from the Directorate of Scientific Research of National Curative and Specialized Stomatology Hospital. Ethical approval was formally obtained from Ghalib University’s Institutional Review Board (IRB Approval #GH-1403-06), in compliance with the Declaration of Helsinki. Informed consent was obtained at the time of treatment for the use of clinical data, and the retrospective use of anonymized records for research purposes was explicitly approved by the IRB. The need for individual consent was waived for anonymized retrospective data. This cross-sectional descriptive study, conducted at the Maxillofacial Surgery Department of the National Curative and Specialized Stomatology Hospital, scrutinized the records of diagnosed patients referred for the extraction of impacted teeth between March 2019 and August 2022. Patient records spanned diverse age groups, with no age restrictions applied. Informed consent was obtained at the time of treatment for use of clinical data, and retrospective use of anonymized patient records for research was approved by the IRB. Data collection involved a 4-year assessment of the archive’s records. Patient lists generated from the archived files and the Health Management Information System (HMIS) were assessed.

The sampling technique used in this study was comprehensive case enumeration. All available dental records from March 2019 to August 2022 were retrieved from the HMIS and physical archives of the Oral and Maxillofacial Surgery Department. Each record was screened for a documented diagnosis of tooth impaction. Every patient with radiographically confirmed impacted teeth during this period was included in the study. No selective or random sampling was employed.

All patients with impacted teeth, regardless of age or gender, who were referred to the National Curative and Specialized Stomatology Hospital in Kabul met the study’s inclusion requirements. Dental records were examined for systemic illness and impacted teeth. A total of 298 cases with impacted teeth were identified. The ages recorded in the patient files ranged from 13 to 83 years, with an average of 25.35 years. Data related to age, gender, systemic diseases, treatments performed, and impacted teeth were recorded. Records were stratified into 5 age groups: <20; 21 to 30; 31 to 40; 41 to 50; and >50. Further investigation of the angulation of impacted teeth was not feasible since radiographs were unavailable, and the records often did not sufficiently describe the angulation of impacted teeth. IBM SPSS Statistics 25 was used for data analysis.

### 2.1. Compliance with the STROBE protocol

This study has been conducted in accordance with the Strengthening the Reporting of Observational Studies in Epidemiology (STROBE) guidelines. The STROBE checklist was followed to ensure comprehensive and transparent reporting of the study’s methodology, results, and discussion.

## 3. Results

A total of 298 patient records with impacted teeth were identified from 4000 screened records (7.5% prevalence). The median age was 24 years (range: 13–83), with 55% of cases occurring in 21 to 30-year-olds. The average age of the patients was 25.35 years, with a range from 13 to 83 years. There were 145 males (48.7%) and 153 females (51.3%) (Table 1). Most patients (93.6%) had at least 1 impacted tooth, while 6% had 2 impacted teeth, and 0.3% had more than 2 impacted teeth. The most common impacted tooth was the lower jaw’s third molar, accounting for 65.1% of cases, followed by the upper jaw’s impacted canine (18.5%) (Table 2). The age group of 21 to 30 years old had the highest incidence of tooth impaction at 55% (Table 3). There was no significant difference in the tendency toward tooth impaction between males (48.7%) and females (51.3%; *P* >.05) (Table 4). Regarding systemic conditions, 2 patients (0.7%) had high blood pressure, 1 patient (0.3%) had diabetes mellitus, and the remaining 295 patients (99%) did not have any systemic diseases. Statistical analysis showed a significant association between age group and the type of impacted tooth, with the third molar being most prevalent in the 21 to 30 age group. The chi-square test for independence indicated no significant gender differences in the prevalence of impacted teeth (*P* >.05).

**Table 1:** Demographic distribution of study participants by gender (n = 298).

		Frequency	Percent	Valid percent	Cumulative percent
Valid	Male	145	48.7	48.7	48.7
	Female	153	51.3	51.3	100.0
	Total	298	100.0	100.0	

**Table 2:** Types and prevalence of impacted teeth with statistical associations (n = 298).

	Tooth	Frequency	Percent	Valid percent	Cumulative percent
Valid	Third maxillary molar	31	10.4	10.4	10.4
	Second maxillary molar	1	0.3	0.3	10.7
	First maxillary molar	1	0.3	0.3	11.1
	First maxillary premolar	10	3.4	3.4	14.4
	maxillary canine	55	18.5	18.5	32.9
	maxillary lateral	1	0.3	0.3	33.2
	Third mandibular molar	194	65.1	65.1	98.3
	Second mandibular molar	2	0.7	0.7	99.0
	First mandibular molar	1	0.3	0.3	99.3
	mandibular canine	2	0.7	0.7	100.0
	Total	298	100.0	100.0	

Chi-square tests revealed significant associations between gender and mandibular third molar impaction (*P* = .002), and between age (21–30 yr) and mandibular third molar impaction (*P* <.001).

**Table 3:** Age group distribution of patients with impacted teeth (n = 298).

	Age	Frequency	Percent	Valid percent	Cumulative percent
Valid	<20	85	28.5	28.5	28.5
	21–30	164	55.0	55.0	83.6
	31–40	32	10.7	10.7	94.3
	41–50	13	4.4	4.4	98.7
	>51	4	1.3	1.3	100.0
	Total	298	100.0	100.0	–

**Table 4:** Frequency distribution of patients by number of impacted teeth (n = 298).

		Frequency	Percent	Valid percent	Cumulative percent
Valid	One impacted	279	93.6	93.6	93.6
	Two impacted	18	6.0	6.0	99.7
	More than 2 impacted	1	0.3	0.3	100.0
	Total	298	100.0	100.0	–

### 3.1. Statistical analysis of risk factors

Chi-square tests identified significant associations between gender and mandibular third molar impaction (χ^2^ = 9.8, *P* = .002), with males having 1.9x higher odds (95% CI: 1.3–2.8) than females. Patients aged 21 to 30 years were 3.2x more likely (95% CI: 2.1–4.9) to present with mandibular third molar impactions compared to other age groups (*P* <.001). Logistic regression confirmed these trends after adjusting for covariates (Table 5). “Table 6 presents the statistical analysis of the types of impacted teeth.” The most common impactions were mandibular third molars, followed by maxillary canines. The angulation of impacted teeth was not evaluated due to the absence of radiographs.

**Table 5:** Multivariate analysis of risk factors for tooth impaction.

Variable	Impacted tooth type	*P*-value	OR	95% CI
**Male versus female**	**Mandibular third molar**	**.002**	**1.9**	**1.3–2.8**
**Age 21–30**	**Mandibular third molar**	**<.001**	**3.2**	**2.1–4.9**
Male versus female	Maxillary canine	.12	1.4	0.9–2.1
**Age 21–30**	**Maxillary canine**	**.03**	**1.8**	**1.1–3.0**

CI = confidence interval, OR = odds ratio.

Significant associations (*P* < .05) in bold.

**Table 6:** Summary of most frequently impacted teeth (n = 298).

Type of impacted tooth	Number of cases	Percentage (%)
Mandibular third molar	208	65.1
Maxillary canine	59	18.5
Other	52	16.4

## 4. Discussion

The purpose of this study was to ascertain the prevalence of impacted teeth in relation to patient age groups, sex, medical history, and age. The results of this investigation are in line with previous research on tooth impaction based on gender. Studies examining the frequency and etiology of dental impaction are numerous, indicating a consistent interest in understanding the factors contributing to this dental issue.

### 4.1. Clinical significance and contribution

*While* impacted teeth are well-studied globally, this is the first investigation of Kabul population, revealing distinct patterns: mandibular third molar prevalence (65.1%) exceeds Asian cohorts like those in Saudi Arabia^[16]^ (40%–50%) and aligns with North African reports^[17]^ (63.8%); maxillary canines show no gender bias (*P* = .12), contrasting with European CBCT studies^[18]^ but resembling some Asian populations. *These* findings suggest regional anatomical or developmental variations, urging tailored prevention strategies in Afghanistan. The clinical significance of this study lies in its potential to inform local dental practices and healthcare policies by providing data specific to this demographic. Unlike many studies that focus on Western or more diverse populations, this research provides insights into a specific geographical and cultural context, contributing valuable data to the global understanding of dental impactions.^[12,19]^ Our *study highlights 2 key trends: Males had 1.9 × higher odds of mandibular third molar impaction than females (P = .002), aligning with hormonal or anatomical hypotheses, and the 21 to 30 age group showed 3.2 × higher likelihood of mandibular impactions (P <.001), consistent with late eruption patterns.*^[19,20]^ *Notably, maxillary canine impactions showed no gender disparity (P = .12), suggesting regional variability in impaction drivers.*^[21–23]^

### 4.2. Comparison with previous studies

The prevalence of third molar impaction in our clinical population (7.5%, 298/4000) represents a conservative estimate compared to radiographic surveys.^[23,24]^ Our mandibular third molar impaction rate (65.1% of cases) aligns with recent global estimates (52%–68%)^[25]^ but exceeds regional averages (58.3% in South Asia).^[26]^ The lower overall prevalence reflects our clinical detection threshold in a hospital setting, contrasting with studies using routine radiographic screening. This variance likely reflects both our inclusive age range (13–83 years) capturing late-developing impactions and potential genetic/dietary influences specific to Kabul population.

The gender disparity in mandibular third molar impactions (OR = 1.8, *P* = .003) mirrors findings from Jordan and North India, where males showed 1.7 to 2.0 × higher prevalence. This consistency across diverse populations strengthens hypotheses about androgen-mediated jaw growth patterns.^[27]^ The pronounced peak in 21 to 30-year-olds (OR = 3.1, *P* <.001) particularly supports the “late eruption theory” – where third molars erupt last amidst already-diminished arch space.^[21,27]^ Notably, our maxillary canine results showed no gender bias (*P* = .12), contrasting with European cohorts but aligning with some Asian studies, suggesting dietary or epigenetic influences on maxillary development.^[7]^

### 4.3. Impaction distribution and patterns

The evaluation of impaction distribution between the maxillary and mandibular bones revealed a higher rate of impactions in the mandible (66.4%) compared to the maxillary (33.6%). The mandibular predominance mirrors recent institutional studies and aligns with biomechanical theories of arch space deficiency, reinforcing that mandibular third molars are more prone to impaction than maxillary counterparts.^[7,19,28]^

### 4.4. Literature updates and expanded discussion

While the current discussion is grounded in significant previous studies, it is essential to incorporate more recent research to provide a comprehensive and updated analysis. Recent 3D analyses corroborate our findings on late-erupting impactions, particularly in the 21 to 30 age group,^[18,29]^ while surgical meta-analyses underscore the need for early intervention in high-risk groups with mandibular third molars.^[2,30]^ These advancements complement earlier work on orthodontic considerations and regional prevalence patterns, offering new mechanistic insights into impaction development and management.^[24,31]^ Collectively, these studies contextualize our Kabul-specific findings within evolving global understanding of dental impactions.

### 4.5. Limitations and strengths

While the hospital-based design of this study may limit its generalizability to the broader population of Kabul, it provides important insight into the clinical burden of impacted teeth in a tertiary care setting. During the 4-year study period, a total of 4000 patients were treated at the Oral and Maxillofacial Surgery Department of the National Curative and Specialized Stomatology Hospital. Among these, 298 individuals were diagnosed with impacted teeth, resulting in a prevalence rate of 7.5% within the department. Although this prevalence may differ from population-based estimates due to referral bias, it reflects a meaningful burden in the clinical population seeking surgical dental care and establishes a foundation for further epidemiological research.

In the context of Kabul dental healthcare system, there are hundreds of private dental clinics operating throughout the city; however, these clinics often lack the infrastructure and resources necessary to manage complex oral surgical procedures such as impacted tooth extractions. There are only 3 governmental dental hospitals in Kabul with full-service facilities, of which the National Curative and Specialized Stomatology Hospital is the primary center for advanced oral and maxillofacial surgeries. Therefore, it serves as the central referral site for complex dental cases across the city and beyond, making it a relevant setting for investigating clinical patterns such as tooth impaction in a hospital-based population.

This study has several important limitations. First, the lack of comprehensive radiographic data precluded analysis of impaction angulation and potentially missed asymptomatic cases, which may affect the generalizability of our prevalence estimates. Second, while our sample size (n = 298) provides meaningful insights into Kabul clinical population, the hospital-based design may not fully represent community-level prevalence. Third, as a retrospective study using anonymized records, individual patient consent was waived by the ethics committee, though all data were de-identified to protect confidentiality. Finally, we were unable to account for potential confounding factors such as nutritional status or orthodontic history due to inconsistent documentation in medical records. Additionally, the records did not consistently document whether the patients had previously undergone dis-impaction procedures, limiting our ability to assess treatment history or recurrence of impactions.

While we identified significant demographic correlations, certain analyses were constrained by unavailable radiographs, precluding assessment of:

1. Impaction angulation’s role in gender/age disparities
2. Arch space measurements to validate mechanical impaction theories
3. Asymptomatic impactions that might alter prevalence estimates

Despite these limitations, the study’s strengths lie in its focused demographic analysis and the comprehensive data collection over a 4-year period, providing a robust dataset for understanding dental impaction patterns in this population.

## 5. Conclusion

This study’s findings indicate that the maxillary third molar, maxillary canine, and mandibular third molar are the teeth most frequently impacted. The mandibular third molar is the most commonly impacted tooth in males, with individuals between the ages of 21 and 30 exhibiting a high impaction rate. The clinical significance of these findings lies in the need for early detection and intervention to prevent potential complications associated with impacted teeth, such as crowding, infection, and cyst formation. It is recommended that dental practitioners consider these prevalence patterns when planning treatment and advising patients. To ascertain the true prevalence of impacted teeth and minimize bias, future studies should include a comprehensive and random sample of the entire population. Radiographic examinations of a specific population should be performed to obtain accurate data on the prevalence, cause, and angulation of impacted teeth. This approach will provide a clearer understanding of the epidemiology of impacted teeth and aid in developing more effective prevention and treatment strategies. The study highlights the importance of addressing impacted teeth in clinical practice and underscores the need for further research to explore the underlying causes and optimal management of these dental anomalies.

## Author contributions

**Conceptualization:** Shahab Uddin Ahmadi, Hedayatullah Ehsan, Sara Wali, Reza Fahimi, Musa Joya, Tamana Barakati, Omid Humkar, Hassina Shadab, Ismail Yaqubi.

**Data curation:** Shahab Uddin Ahmadi, Hedayatullah Ehsan, Yahya Fayaz, Sara Wali, Reza Fahimi, Musa Joya, Tamana Barakati, Omid Humkar, Hassina Shadab, Ismail Yaqubi.

**Formal analysis:** Shahab Uddin Ahmadi, Hedayatullah Ehsan, Yahya Fayaz, Sara Wali, Reza Fahimi, Musa Joya, Tamana Barakati, Omid Humkar, Hassina Shadab, Ismail Yaqubi.

**Investigation:** Shahab Uddin Ahmadi, Hedayatullah Ehsan, Yahya Fayaz, Musa Joya, Tamana Barakati, Omid Humkar, Hassina Shadab, Ismail Yaqubi.

**Methodology:** Shahab Uddin Ahmadi, Hedayatullah Ehsan, Yahya Fayaz, Reza Fahimi, Musa Joya, Tamana Barakati, Omid Humkar, Hassina Shadab, Ismail Yaqubi.

**Project administration:** Shahab Uddin Ahmadi, Hedayatullah Ehsan, Yahya Fayaz, Reza Fahimi, Tamana Barakati, Omid Humkar, Hassina Shadab, Ismail Yaqubi.

**Resources:** Hedayatullah Ehsan, Yahya Fayaz, Sara Wali, Reza Fahimi, Tamana Barakati, Ismail Yaqubi.

**Software:** Shahab Uddin Ahmadi, Hedayatullah Ehsan, Sara Wali, Reza Fahimi.

**Supervision:** Hedayatullah Ehsan, Tamana Barakati, Omid Humkar.

**Validation:** Hedayatullah Ehsan, Reza Fahimi.

**Visualization:** Hedayatullah Ehsan, Musa Joya.

**Writing – original draft:** Shahab Uddin Ahmadi, Hedayatullah Ehsan, Yahya Fayaz, Sara Wali, Reza Fahimi, Musa Joya, Tamana Barakati, Omid Humkar, Hassina Shadab, Ismail Yaqubi.

**Writing – review & editing:** Hedayatullah Ehsan, Yahya Fayaz, Sara Wali, Reza Fahimi, Musa Joya, Tamana Barakati, Omid Humkar, Hassina Shadab, Ismail Yaqubi.
